# FgBud3, a Rho4-Interacting Guanine Nucleotide Exchange Factor, Is Involved in Polarity Growth, Cell Division and Pathogenicity of *Fusarium graminearum*

**DOI:** 10.3389/fmicb.2018.01209

**Published:** 2018-06-07

**Authors:** Chengkang Zhang, Zenghong Luo, Dongdong He, Li Su, Hui Yin, Guo Wang, Hong Liu, Christopher Rensing, Zonghua Wang

**Affiliations:** ^1^Institute of Environmental Microbiology, College of Resources and Environment, Fujian Agriculture and Forestry University, Fuzhou, China; ^2^College of Life Science, Fujian Agriculture and Forestry University, Fuzhou, China; ^3^College of Plant Protection, Fujian Agriculture and Forestry University, Fuzhou, China; ^4^J. Craig Venter Institute, La Jolla, CA, United States

**Keywords:** *Fusarium graminearum*, RhoGEF, *FgBUD3*, septum formation, pathogenicity

## Abstract

Rho GTPases are signaling macromolecules that are associated with developmental progression and pathogenesis of *Fusarium graminearum*. Generally, enzymatic activities of Rho GTPases are regulated by Rho GTPase guanine nucleotide exchange factors (RhoGEFs). In this study, we identified a putative RhoGEF encoding gene (*FgBUD3*) in *F. graminearum* database and proceeded further by using a functional genetic approach to generate *FgBUD3* targeted gene deletion mutant. Phenotypic analysis results showed that the deletion of *FgBUD3* caused severe reduction in growth of *FgBUD3* mutant generated during this study. We also observed that the deletion of *FgBUD3* completely abolished sexual reproduction and triggered the production of abnormal asexual spores with nearly no septum in *ΔFgbud3* strain. Further results obtained from infection assays conducted during this research revealed that the *FgBUD3* defective mutant lost its pathogenicity on wheat and hence, suggests FgBud3 plays an essential role in the pathogenicity of *F. graminearum*. Additional, results derived from yeast two-hybrid assays revealed that FgBud3 strongly interacted with FgRho4 compared to the interaction with FgRho2, FgRho3, and FgCdc42. Moreover, we found that FgBud3 interacted with both GTP-bound and GDP-bound form of FgRho4. From these results, we subsequently concluded that, the Rho4-interacting GEF protein FgBud3 crucially promotes vegetative growth, asexual and sexual development, cell division and pathogenicity in *F. graminearum*.

## Introduction

Fusarium head blight (FHB), a disease of wheat and barley is mainly caused by the filamentous ascomycete *Fusarium graminearum* (teleomorph *Gibberella zeae*) (Goswami and Kistler, 2004). Besides causing huge yield and economic losses, the FBH fungus also secretes a variety of harmful mycotoxins, including, deoxynivalenol (DON), zearalenone and T-2 toxin, into infested grains (Desiardins et al., 1996; Bottalico and Perrone, 2002; Proctor et al., 2002; Goswami and Kistler, 2004; Starkey et al., 2007).

Collectively, Rho GTPases are a small group of GTPases in the Ras GTPase superfamily. They switch between active and inactive form by binding to GTP and GDP respectively. The activation of Rho GTPases by guanine nucleotide-exchange factors (GEFs) transforms it from GDP binding state to GTP binding state (Iden and Collard, 2008). In the fungal kingdom, Rho GTPases including Rho1 and Cdc42 and their corresponding GEF activators have been well studied in budding yeast. For instance, research has shown that in *Saccharomyces cerevisiae* three RhoGEFs; Rom1, Rom2 and Tus1 play coordinated roles in activating Rho1 which is an essential GTPase associated within the cell wall integrity pathway, actin ring assembly and cytokinesis processes (Ozaki et al., 1996; Manning et al., 1997; Schmidt et al., 1997; Schmelzle et al., 2002; Yoshida et al., 2006, 2009). Loss of Tus1 or Rom1 alone resulted in only subtle phenotypes whereas loss of Rom2 caused cell lysis at high temperatures (Schmelzle et al., 2002; Lesage et al., 2005; Hillenmeyer et al., 2008). Rom1 and Rom2 perform overlapping functions, hence, loss of both of genes had a lethal effect and caused cell lysis at all temperatures (Ozaki et al., 1996). Contrary to previous suggestions that Cdc24 functions as the sole RhoGEF for Cdc42, current investigations interestingly identified Bud3, a protein containing a putative GTP-binding motif and a Dbl homology (DH) domain (also known as RhoGEF domain), as additional RhoGEF for Cdc42 and further proceeded to show that Bud3 functions as Cdc42 GEF during early G1 phase in budding yeast (Sloat et al., 1981; Adams et al., 1990; Chenevert et al., 1994; Zheng et al., 1994; Kang et al., 2014). Previous studies also identified Bud3 homologues in *Neurospora crassa* and *Aspergillus nidulans* as Rho4 GEFs (Justa-Schuch et al., 2010; Si et al., 2010). Insights gained from studies conducted in yeast and filamentous fungi showed that some of these Rho GTPases, i.e., Rho1 in the budding yeast, could be activated by more than one GEF (Justa-Schuch et al., 2010; Krause et al., 2012; Kang et al., 2014). Additional evidence also confirmed that a given GEF could regulate two different Rho GTPases. For example, it has been shown that Cdc24, besides operating as GEF for Cdc42, also activated GEF for Rac1 in *N. crassa* (Araujo-Palomares et al., 2011). Normally, activated Rho GTPases interact with downstream effectors on membranes to regulate signal transduction pathways (Iden and Collard, 2008). For instance, FgRac1 specifically interacted with downstream targets including FgCla4 and FgNoxR to regulate asexual and sexual development in *F. graminearum*, respectively (Zhang et al., 2013, 2016). In some plant pathogens, Rho GTPases were not only important for polarity growth, sexual and asexual reproduction, cytokinesis but also pathogenesis (Zheng et al., 2007, 2009; Chen et al., 2008; Harris, 2011; Kwon et al., 2011; Nesher et al., 2011; Zhang et al., 2013). Up to now, the functions of RhoGEFs and their relationship with different Rho GTPases in plant pathogens are still less studied. Therefore, it is important to identify their roles in fungal development and pathogenesis process in plant pathogens.

In our previous study, we identified six Rho GTPases in *F. graminearum*, and showed that all six Rho GTPases were associated with development and pathogenesis of *F. graminearum* in varying manner (Zhang et al., 2013). For example, we demonstrated that deletion of FgCdc42 caused serious impairment in, growth, conidiation, sexual development and rendered resultant mutant strains non-pathogenic, while a deletion of FgRho2 only caused a slight reduction in growth and virulence (Zhang et al., 2013). However, the influence of GEF proteins on regulatory activities of Rho GTPases in *F. graminearum* has not been reported. In this study, we identified six putative RhoGEF proteins in *F. graminearum* by homology alignment. We further characterized functions of a Rho4 interacting RhoGEF protein, FgBud3, and found it was not only important for vegetative growth, reproduction and pathogenicity, but also for cell division in *F. graminearum*.

## Materials and Methods

### Strains, Media and Growth Condition

Conidia of the *FgBUD3* deletion mutants, Δ*Fgbud3-1* and Δ*Fgbud3-6*, were stored in 20% glycerol solution at -80°C. Complete medium (CM) and synthetic low-nutrient agar (SNA) medium was used for mycelial growth assays and conidiation assays as previously described (Zheng et al., 2012). Strains grown on CM plates supplemented with 0.1 mg/mL Calcofluor White (CFW) were used to test the sensitivity against cell-wall-disrupting agents (Jiang et al., 2011). Sexual reproduction assays were performed on carrot agar (CA) medium in accordance with a previously described experimental protocol (Bowden and Leslie, 1999; Zheng et al., 2013).

### Generation of the *FgBUD3* Deletion Mutants and the Complementary Strain

To generate an *FgBUD3* deletion vector construct, we first use primer pairs AF-BUD3/AR- BUD3 and BF- BUD3/BR- BUD3 (**Supplementary Table S1**) to amplify the upstream and downstream fragments of the *FgBUD3* gene from the genome of *F. graminearum* wild-type strain PH-1, the resulting amplicons were cloned by linking upstream and downstream sequences of hygromycin-resistance gene in a pCX62 vector respectively. The protoplasting buffer [0.5 g driselase (D9515, Sigma-Aldrich, Inc.), 0.1 g lysing enzymes (L1412, Sigma-Aldrich, Inc.) and 20 mL 1 M KCl solution] was used for protoplast preparation of *F. graminearum*. The details of the protoplast preparation and fungal transformation were described in an established protocol (Hou et al., 2002). Hygromycin-resistant transformants were screened by PCR and RT-PCR with primer pair OF- BUD3/OR- BUD3 and further verified by Southern blot.

For complementation of the *FgBUD3* deletion, a fragment amplified by primer pairs CF-BUD3/CR-BUD3 (**Supplementary Table S1**) was co-transformed with a geneticin-resistant gene fragment into the protoplast of the *FgBUD3* deletion mutant (Δ*Fgbud3-1*). Geneticin-resistant transformants were screened by PCR and RT-PCR.

### Infection and DON Production Assays

For flowering wheat heads (Bainong 979) infection, conidia were collected from 7-day-old SNA plates and resuspended in sterile distilled water to a concentration of 2 × 10^5^ conidia/ml. The middle spikelet of wheat flowers was inoculated with 10 μl of the conidial suspension as described (Gale et al., 2002; Wang et al., 2012). Autoclaved rice grains were inoculated with conidia and cultured for 2 weeks and assayed for DON toxin as described (Seo et al., 1996). Ergosterol levels were used to normalize DON content per fungal mass. The DON production level was also detected by the DON Plate Kit (Shenzhen Finder Biotech Co., Ltd.), 10^4^ conidia of each strain were grown in 1.5 ml liquid TBIA culture (Zhang et al., 2016). Fifty microliter of 8-old-day cultures or cultures with 10-fold or 1000-fold dilutions were used for DON detection following the manipulation instructions of kit. The thorough dried mycelia weight of each strain in the cultures was used to normalize the DON production level.

### Staining and Microscopy Observation

Conidia or mycelia were stained with 10 μg/mL CFW (Rasmussen and Glass, 2007) and 5 μg/mL 4′,6-diamidino-2-phenylindole (DAPI) (Seong et al., 2008) for septa and nuclei observation, respectively. An Olympus BX51 Microscope was used to perform microscopic observations. The morphology of aerial hypha, conidia and germinated conidia of different strains were observed under light microscopy. The septa and nuclei observation was performed under UV microscopy. The microscopy images were used to observe the morphology of conidia and hypha and to calculate the quantity of septa and nuclei. We randomly chose more than 100 conidia of each strain to calculate the length and width of conidia and the quantity of septa or nuclei.

### Yeast Two-Hybrid Assay

Yeast two-hybrid assay was performed using the MATCHMAKER GAL4 Two-Hybrid System 3 (Clontech). FgRho4 ORF amplicon was amplified with primer pairs BDF-RHO4/BDR-RHO4 from cDNA of PH-1 with the site mutation C267S to ensure that the FgRho4 protein cannot be prenylated and was thus soluble (Chen et al., 2008). The amplicon was cloned into the pGBKT7 vector to create the vector BD-FgRho4. Similar methods were used to construct the vectors BD-FgRho1, BD-FgRho2, BD-FgRho3, BD-FgRac1 and BD-FgCdc42. Template BD-FgRho4 vector and two primers CAF and CAR (**Supplementary Table S1**) were used to generate a constitutively active (G64V) mutation with primers containing the substitution of the glycine (G64V) of FgRho4 with valine. Primers DNF and DNR (**Supplementary Table S1**) were used to generate dominant negative mutations, the (D172A) mutation with primers containing the substitution of the aspartic acid (D172A) with alanine. The resultant vectors were named BD-FgRho4 (CA) and BD-FgRho4 (DN). A partial *FgBUD3* ORF including RhoGEF domain region was amplified from cDNA of PH-1 with primer pairs ADF-BUD3/ADR-BUD3, and the amplicon was cloned into pGADT7 vector to create AD-FgBud3 vector. The resultant bait and prey vectors were confirmed by sequencing and were co-transformed in pairs into the yeast strain AH109 (Schiestl and Gietz, 1989). The plasmid pairs, pGBKT7-53 and pGADT7 and pGBKT7- Lam and pGADT7-T, served as the positive and the negative control, respectively. The isolation and confirmation of transformants were described previously (Chen et al., 2008).

## Results

### Identification of RhoGEF Proteins in *F. graminearum*

**FIGURE 1:**
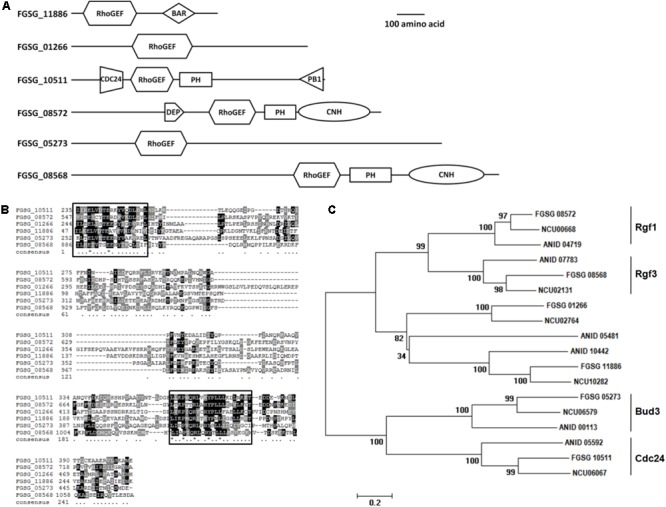
*Fusarium graminearum* encodes six putative RhoGEF proteins. **(A)** The domain distribution of six *F. graminearum* RhoGEFs. **(B)** The RhoGEF domain amino acid sequences of six *F. graminearum* RhoGEFs were aligned. Residues conserved in all proteins were highlighted in black or gray shaded. The black frames showed the conserved regions. **(C)** Phylogenetic comparison of six *F. graminearum* RhoGEFs with other fungal RhoGEFs. The amino acid sequences of RhoGEFs from *N. crassa* (NCU), *A. nidulans* (ANID) and *F. graminearum* (FGSG) were analyzed by the Clustal X 1.83 program to create a progressive alignment dendrogram. The branch lengths were proportional to the mean number of differences per residue along each branch. Accession number of each protein was shown as follows: FGSG_08572, XP_011320178.1; NCU00668, XP_965808.3; ANID_04719, XP_662323.1; ANID_07783, XP_681052.1; FGSG_08568, XP_011320182.1; NCU02131, XP_011392943.1; FGSG_01206, XP_011317046.1; NCU02764, XP_964259.2; ANID_05481, XP_663085.1; ADID_10442, XP_661358.1; FGSG_11886, XP_011316798.1; NCU10282, XP_001728403.1; FGSG_05273, XP_011323784.1; NCU06579, XP_961939.3; ANID_00113, XP_657717.1; ANID_05592, XP_663196.1; FGSG_10511, XP_011319503.1; NCU06067, XP_959830.3.

We identified six proteins harboring RhoGEF domains in the *F. graminearum* genome database^1^ encoded by, FGSG_08572, FGSG_08568, FGSG_01266, FGSG_11886, FGSG_05273, and FGSG_10511. These proteins differed in lengths ranging from 582 to 1704 amino acids. In addition to the RhoGEF domain, four of these proteins (FGSG_08572, FGSG_8568, FGSG_11886, and FGSG_10511) contained additional domains, which include, BAR, PB1, CDC24 domains and thus, suggested these four proteins may assume diverse functions in *F. graminearum* (**Figure 1A**). Subsequent alignments performed with amino acid sequences of these six putative *F. graminearum* RhoGEFs recorded very low homology between the respective GEF proteins. However, domain alignment results showed that seven residues in two of the motifs identified are only conserved in the RhoGEF domain sequences of the six proteins (**Figure 1B**). These results suggested these seven amino acids might be important for the function of RhoGEF proteins.

We also initiated a comparative homology search by comparing these *F. graminearum* RhoGEFs proteins identified in this study with RhoGEF proteins previously reported in *N. crassa* and *A. nidulans* (Justa-Schuch et al., 2010; Si et al., 2010; Araujo-Palomares et al., 2011; Richthammer et al., 2012). This search found homologs of *F. graminearum* RhoGEF proteins in these two fungi. Maximum likelihood analysis of these RhoGEF proteins revealed six independent lineages for each RhoGEF member in *N. crassa, A. nidulans*, and *F. graminearum* (**Figure 1C**). From these integrated analyses, we successfully identified four *F. graminearum* RhoGEF proteins in accordance with their homologs in *N. crassa* and *A. nidulans* and named them FgBud3, FgRgf3, FgRgf1, and FgCdc24, which encoded by FGSG_05273, FGSG_08568, FGSG_08572, and FGSG_10511, respectively (Justa-Schuch et al., 2010; Si et al., 2010; Araujo-Palomares et al., 2011; Richthammer et al., 2012). One of these *F. graminearum* RhoGEFs, FgBud3, was further characterized in this study.

### The Deletion of *FgBUD3* Exerted Serious Adverse Effect on Vegetative Growth of Δ*Fgbud3* Strain

FgBud3 contains 1477 amino acids and a RhoGEF domain at the N-terminus (**Figure 1B**). To study the function of FgBud3, we generated *FgBUD3* deletion mutants by replacing the ORF of *FgBUD3* with a hygromycin resistance gene as a selectable marker in *F. graminearum* and confirmed the generated gene deletion by PCR, RT-PCR and Southern blot assay (**Supplementary Figure S1**).

**FIGURE 2:**
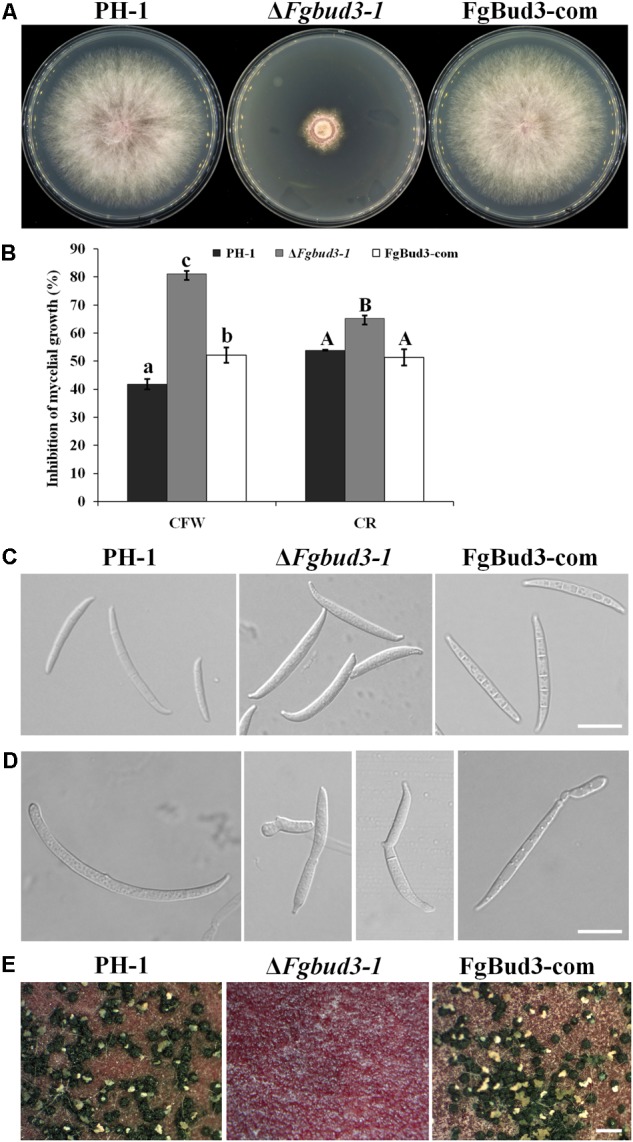
The hyphal growth, asexual and sexual developmental defects of the *FgBUD3* deletion mutant. **(A)** The hyphal growth of strains grown on CM plates. **(B)** Sensitivity of each strain to 100 μg/mL CFW and 1.5 mg/mL CR (Congo red). Different letters were used to mark statistically significant difference (*P* < 0.05). **(C)** Conidia produced by PH-1, Δ*Fgbud3-1* and FgBud3-com with normal Fusarium-shape. Bar = 20 μm. **(D)** Conidia produced by the *FgBUD3* deletion mutant with different kinds of abnormal shape. Bar = 20 μm. **(E)** The sexual development defect of the *FgBUD3* deletion mutant. Bar = 500 μm. PH-1, wild-type strain; Δ*Fgbud3-1*, gene *FgBUD3* deletion mutants; FgBud3-com, complemented strain of *FgBUD3* deletion mutant.

Two of the *FgBUD3* deletion mutants (Δ*Fgbud3-1* and Δ*Fgbud3-6*) and the wild-type strain were grown on CM plates for 4 days. The *FgBUD3* deletion mutants grew much slower than the wild-type (**Table 1** and **Figure 2A**). Compared with the dense aerial hypha produced by the wild-type strain PH-1, Δ*Fgbud3-1* only produced tiny aerial hypha (**Figure 2A**). We further generated a complemented strain (FgBud3-com) by reintroducing the native *FgBUD3* into Δ*Fgbud3-1* strain and normal vegetative growth phenotype was observed (**Table 1** and **Figure 2A**). The *FgBUD3* deletion mutant grew very poorly on CM plates containing 100 μg/mL CFW or 1.5 mg/mL Congo red. The sensitivity of Δ*Fgbud3-1* to CFW or Congo red is higher than sensitivity of PH-1 and FgBud3-com (**Figure 2B**). Moreover, many protoplasts could be observed after mycelia lysed after incubation in protoplasting buffer for 1 h (data not shown) in the *FgBUD3* deletion mutant. In contrast, much fewer protoplasts of the wild-type strain could be observed under the same treatment, suggesting the *FgBUD3* deletion mutant was highly sensitive to cell wall damaging agents. These results suggested FgBud3 was involved in polarity growth and cell wall integrity.

**Table 1:** Phenotype characterization of *FgBUD3* deletion mutants of *F. graminearum*.

Strain	Growth rate (mm/day)^α^	Conidiation				DON/Erg^𝜀^	DON (ppb)^ζ^	Disease index^η^
		Conidium quantity (× 10^4^/cm2)^β^	Conidium length (μm)^γ^	Conidium width (μm)^γ^	Nucleus quantity per conidium^δ^			
PH-1	16.4 ± 0.5^a∗^	6.9 ± 0.4^a^	45.64 ± 9.90^a^	4.75 ± 0.77^a^	5.05 ± 1.18^a^	0.459 ± 0.142^a^	1600.4 ± 401.4^b^	20.3 ± 7.6^a^
Δ*Fgbud3-1*	4.4 ± 0.3^b^	2.6 ± 1.1^c^	56.00 ± 21.21^b^	5.03 ± 0.79^b^	10.63 ± 3.44^b^	0.002 ± 0.001^b^	non-detectable^∗∗^	0.0 ± 0.0^b^
Δ*Fgbud3-6*	4.0 ± 0.6^b^	–	–	–	–	–	–	0.0 ± 0.0^b^
FgBud3-com	17.4 ± 1.4^a^	5.7 ± 0.4^b^	45.85 ± 10.48^a^	4.73 ± 0.57^a^	4.92 ± 1.11^a^	–	6330.0 ± 240.2^a^	20.0 ± 7.4^a^

*^α^Growth rate was measured after incubating on CM agar plates for 4 days. ^β^Conidium quantity was measured by counting the number of conidia divided by colony area collected from 5-day-old SNA plates. ^γ^More than 100 conidia of each strain were chosen randomly to measure the length and width. ^δ^More than 100 conidia of each strain were stained by DAPI were chosen randomly to measure the nucleus quantity. ^𝜀^DON/Ergosterol ratio was determined with 2-week-old rice grain cultures. Ergosterol was measured to quantify fungal biomass. ^ζ^DON concentration was determined with 8-day-old mycelia in TBIA culture. Mycelia weight was measured to quantify fungal biomass. ^η^Disease was rated by the number of symptomatic spikelets 14 days after inoculation. ^∗^Mean and standard error were calculated from at least three independent measurements and were analyzed with the *T*-test. The same letter indicated that there was no significant difference. Different letters were used to mark statistically significant difference (*P* < 0.05). ^∗∗^Lower than the detection limits.*

### FgBud3 Is Required for Conidiogenesis and Sexual Reproduction

We incubated the *FgBUD3* deletion mutant and the wild-type strain on SNA plates for 5 days, and observed that the mutant produced fewer conidia than the wild-type strain (**Table 1**). We also noticed that the average conidium size of Δ*Fgbud3-1* was larger than PH-1 and FgBud3-com; many conidia of Δ*Fgbud3-1* were deformed in shape, for example, some these conidia conjugated with the phialide cell (**Table 1** and **Figures 2C,D**). From these results, we inferred that FgBud3 is involved in the regulation of the conidia morphogenesis process in *F. graminearum*.

Our investigations further showed that deletion of *FgBUD3* in the homothetic fungus, *F. graminearum* completely abolished perithecia production in the Δ*Fgbud3-1* strain cultured on CA plates after induction, indicating that FgBud3 is essential for sexual reproduction in *F. graminearum* (**Figure 2E**).

### FgBud3 Is Involved in Cell Division

**FIGURE 3:**
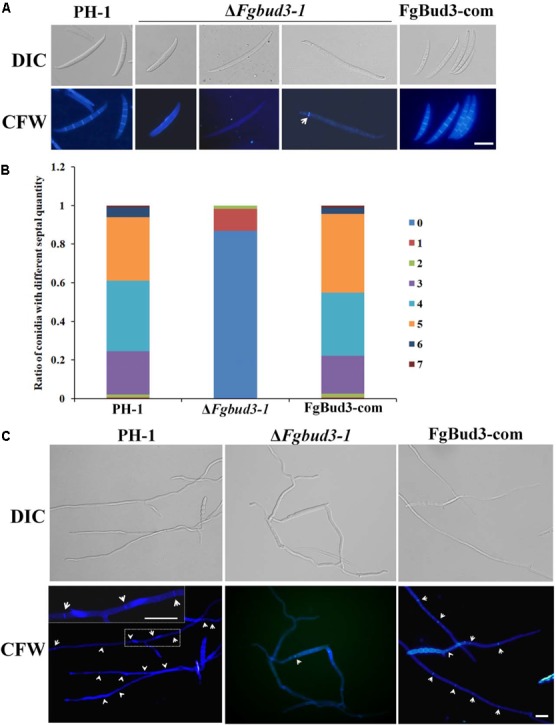
The septum defective phenotype of the *FgBUD3* deletion mutant. **(A)** Conidia of PH-1, Δ*Fgbud3-1* and FgBud3-com were stained with 1 μg/ml CFW and examined by microscopy under DIC or UV light. **(B)** Ratio of conidia with different septal quantity of each strain. **(C)** Hypha of each strain were stained and examined. The arrows indicate septa. Bar = 20 μm.

**FIGURE 4:**
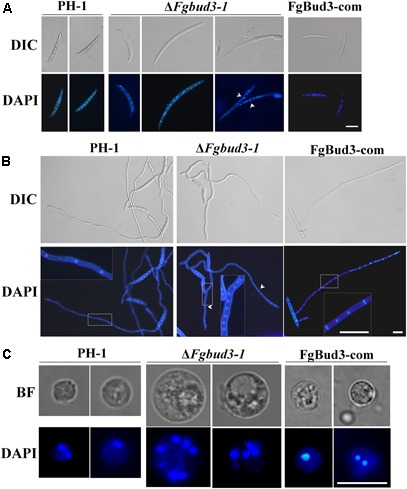
The nuclear division phenotype of the *FgBUD3* deletion mutant. Conidia **(A)** or hyphae **(B)** of PH-1, Δ*Fgbud3-1* and FgBud3-com were stained with 4′,6-diamidino-2-phenylindole (DAPI) and examined by microscopy under DIC or UV light. The arrows indicate septa. Bar = 20 μm. **(C)** Protoplasts of each strain were stained and examined. Bar = 10 μm.

Deletion of *BUD3* in *A. nidulans* and *N. crassa* resulted in defective septum formation (Justa-Schuch et al., 2010; Si et al., 2010). In accordance with this report, we hypothesized that FgBud3 might play a similar role in *F. graminearum*. To test this hypothesis, we stained conidia of the *FgBUD3* deletion mutant and wild-type strain with CFW. Corresponding results obtained from these assays showed that most conidia produced by Δ*Fgbud3-1* lacked septa while the majority of conidia produced by the wild-type strain possessed 3–5 septa (**Figures 3A,B**). In some rare cases, we observed 1–2 septa in some conidia produced by the deletion mutant (**Figures 3A,B, 4A**). In addition, we used DAPI to perform conidia nuclei staining. Results from these examinations revealed that conidia produced by the Δ*Fgbud3-1* strain contain more nuclei than conidia obtained from the wild-type or complemented strain (**Table 1** and **Figure 4A**). Similar results were observed in fresh hypha germinating from conidia after incubation in CM culture for 6 h (**Figures 3C, 4B**). Moreover, protoplasts from Δ*Fgbud3-1* generated in protoplasting buffer were much bigger and harbored more nuclei than protoplasts of the wild-type or the complemented strain (**Figure 4C**). These results indicated the FgBud3 play key role in cell division related processes.

### FgBud3 Is Important for Pathogenicity and DON Production

**FIGURE 5:**
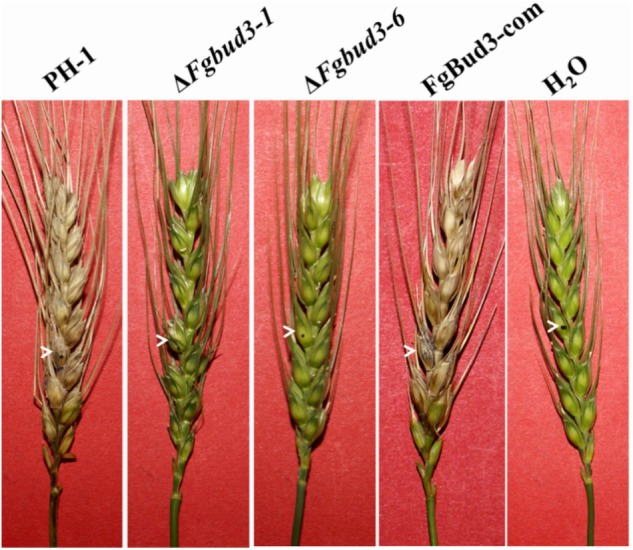
Infection assays with flowering wheat heads. Wheat heads infected with conidia of PH-1, Δ*Fgbud3-1* and FgBud3-com. The arrows indicate inoculated spikelets.

We inoculated a spikelet in the middle of wheat heads with conidia of the *FgBUD3* deletion mutants, wild-type strain or the complemented strain. After 14 days, serious symptoms were observed in wheat heads inoculated with conidia obtained from the wild-type strain or the complemented strain (**Table 1** and **Figure 5**). On the contrarily, no symptoms were observed in wheat head spikelets inoculated with conidia harvested from the Δ*Fgbud3-1* strain, indicating that FgBud3 is required for pathogenicity of *F. graminearum* (**Table 1** and **Figure 5**). Furthermore, we monitored the level of DON generated in the Δ*Fgbud3-1* strain compared to the wild-type strain in rice grains and TBIA culture. DON production assessment results showed that, the level of DON generated in both rice grains and TBIA mudium inoculated with Δ*Fgbud3-1* strain was at a significantly lower than that inoculated with wild-type strain, suggesting that FgBud3 played an important role in regulating DON production *F. graminearum* (**Table 1**).

### FgBud3 Interacts With Both GDP-Bound and GTP-Bound FgRho4

**FIGURE 6:**
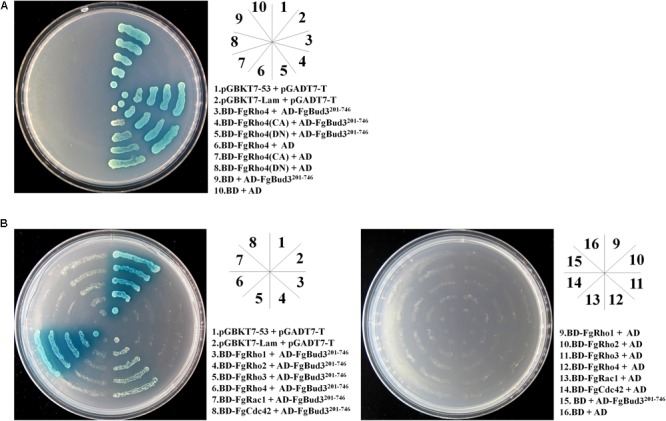
FgBud3 interacts with FgRho4. **(A)** Yeast two-hybrid assay with FgRho4 (3) or FgRho4-CA (4) or FgRho4-DN (5) as the bait and FgBud3^201-746^ (3–5) as the prey. **(B)** Yeast two-hybrid assay with each FgRho GTPase (3–8) as the bait and FgBud3^201-746^ (3–8) as the prey. Twenty microliter yeast cells (10^7^ cell/mL) of each yeast transformants were grown on the SD-Leu-Trp-His-Ade plates were assayed for α-galactosidase activity. The interaction of pGBKT7-53 and pGADT7-T (1) was used as the positive control. The other interactions were used as the negative control.

Bud3-homologs were identified as Rho4GEFs in both *N. crassa* and *A. nidulans*, FgBud3 could play a similar role in *F. graminearum* (Justa-Schuch et al., 2010; Si et al., 2010). As a putative Rho4GEF, FgBud3 would be predicted to interact with an inactivated (GDP-bound) FgRho4 to replace GTP for GDP. Thus, a yeast two-hybrid assay was performed between RhoGEF domain of FgBud3 and different states of FgRho4. The result indicated the FgBud3 RhoGEF domain interacted with wild-type FgRho4 and both GTP-bound (FgRho4-CA) and GDP-bound FgRho4 (FgRho4-DN) (**Figure 6A**). Furthermore, we performed another yeast two-hybrid assay to determine if FgBud3 interacted with other Rho GTPases in *F. graminearum*. The result indicated the FgBud3 RhoGEF domain interacted not only with FgRho4 but also with FgRho2, FgRho3 and FgCdc42. However, the interaction between the FgBud3 RhoGEF domain and FgRho4 was stronger than other interactions (**Figure 6B**).

## Discussion

The exchange of GDP for GTP is required for the activation of Rho GTPases and RhoGEF proteins are responsible for this process. Rho GTPases in *F. graminearum* were shown to be important to fungal development and/or pathogenesis, but their activators, RhoGEF proteins, had not been reported yet (Zhang et al., 2013). In this study, we identified six putative RhoGEF proteins in *F. graminearum*. In *N. crassa*, a RhoGEF, Cdc24, regulated two Rho GTPases (Cdc42 and Rac1) while Rho4 was activated by two RhoGEFs (Bud3 and Rgf3) (Justa-Schuch et al., 2010; Araujo-Palomares et al., 2011). It suggested the relationship between RhoGEFs and Rho GTPases was not “one RhoGEF to one Rho GTPase.” This pattern may be also occurring in another filamentous fungus, *F. graminearum*. Homologs of Cdc24, Bud3, Rgf3 and Rgf1 in *N. crassa* and *A. nidulans* play important roles in polarity growth, conidiation and/or septation suggesting *F. graminearum* RhoGEFs could play similar roles (Justa-Schuch et al., 2010; Si et al., 2010; Araujo-Palomares et al., 2011; Richthammer et al., 2012). In addition, they could have some different functions, e.g., a function in pathogenesis, as compared to homologs in *N. crassa* and *A. nidulans*. Therefore, we characterized the functions of one of the four RhoGEF proteins, FgBud3, in *F. graminearum*. Our results further demonstrated FgBud3 is involved in multiple processes, such as polarity growth, conidiogenesis, sexual reproduction, cell division and pathogenicity.

Bud3-homolog has been first studied in a plant pathogen in our study, and the result indicated FgBud3 was essential for pathogenicity in *F. graminearum* (**Table 1** and **Figure 5**). The serious growth defect is one of the reasons accounting for pathogenicity defects displayed by the *FgBUD3* deletion mutant as shown in many other mutants of *F. graminearum* with growth defects, such as deletion mutants of some transcription factors or kinases (Son et al., 2011; Wang et al., 2011). The deletion of these types of genes usually does not only exert serious effect on growth, but also interferes with cell wall sensitivity, triggers reduction in DON production and/or renders respective deletion mutants non-pathogenic. Although, the growth of FgRho2 deletion mutant was indistinguishable from the wild-type strain, it however, displayed attenuated virulence and compromised cell wall sensitivity (Zhang et al., 2013). The deletion mutants of some *TRI* genes such as *TRI6* and *TRI10* did not display any DON production and subsequently almost no virulence without having any other defects suggesting this toxin was a key factor for pathogenesis (Seong et al., 2009). Thus, it comes as no surprise that the *FgBUD3* deletion mutant with sensitive cell wall and almost no DON production is also non-pathogenic.

The *FgBUD3* deletion mutant showed multiple defects compared to wild-type, and the complemented strain FgBud3-com was successful to recover these defects. Interestingly, FgBud3-com produced four times higher DON than wild-type PH-1. We can reason that the high DON production level of the complemented strain was due to the random insertion of *FgBUD3* in the complemented strain which could cause a different expression pattern of *FgBUD3* compare to the wild-type.

Depletion of FgBud3 resulted in serious defects in aerial hyphal growth and abnormal conidiation suggesting FgBud3 is required for polarity growth and maintenance. Fungal Rho GTPases are well known for regulating polarity growth and maintenance (Harris, 2011). In *F. graminearum*, loss of *FgRHO4* caused serious conidiogenesis defects such as producing many abnormal conidia, and some conidia could even be generated on other conidia (Zhang et al., 2013). FgRac1 and FgCdc42, two other Rho GTPases, were also shown to be important for polarity growth and maintenance in that their corresponding gene deletion mutant led to a hyper-branching phenotype and an abnormal conidia shape, respectively (Zhang et al., 2013). The PAK kinase FgCla4, a downstream target of both FgCdc42 and FgRac1, was also required for polarity growth (Zhang et al., 2013). These results suggested FgBud3 might be involved in activating at least one of these Rho GTPases to regulate polarity growth by interacting with their downstream targets. In the Rho GTPase family, Rho4 is well conserved and regulates septation in filamentous fungi (Rasmussen and Glass, 2005, 2007; Justa-Schuch et al., 2010; Si et al., 2010; Kwon et al., 2011; Zhang et al., 2013). In *F. graminearum*, FgRho4 is required for both septum formation and nuclear division (Zhang et al., 2013). FgBud3, a putative RhoGEF, exerted similar functions, not only on septum formation and nuclear division but also on vegetative growth, cell wall integrity, conidiogenesis, sexual reproduction and pathogenesis in a manner similar to FgRho4. These results strongly imply FgBud3 to be a Rho4GEF for FgRho4. A yeast two hybrid assay was performed to investigate the interaction between FgBud3 and FgRho4. Unfortunately, the interaction was negative. We supposed it was because the large size of the FgBud3 protein (1477 amino acids) and it was difficult to make a correct protein folding in yeast. Therefore, we used the RhoGEF domain to replace the full length of FgBud3 as a prey in the yeast two hybrid assay. The result revealed the interaction between an inactivated form (GDP-bound) of FgRho4 and FgBud3 indicating FgBud3 was a GEF of FgRho4 (**Figure 6A**).

In *N. crassa*, the RhoGEF Cdc24 can activate two Rho GTPases, Rac1 and Cdc42 (Araujo-Palomares et al., 2011). Thus, we hypothesized that FgBud3 might interact with other Rho GTPases to regulate their downstream targets in *F. graminearum*. To test this hypothesis, one more yeast two hybrid assay was exerted and the result showed the RhoGEF domain of FgBud3 also interacted with FgRho2, FgRho3, and FgCdc42, though the intensity of these interactions were weaker than the interaction between FgBud3 and FgRho4 (**Figure 6B**). In *N. crassa*, Bud3 was a specific RhoGEF of Rho4 (Justa-Schuch et al., 2010). If the pattern was also happening in *F. graminearum*, it seemed the weak interactions between partial of FgBud3 and other Rho GTPases, e.g., FgRho3, may not be accurate enough due to the compromised specificity of FgBud3. However, the interaction results were still reliable, because the RhoGEF domain of FgBud3 did not interact with FgRho1 and FgRac1 (**Figure 6B**). The recent result showed Bud3 activated Cdc42 to establish a proper growth site in budding yeast (Kang et al., 2014), suggesting FgBud3 could also be an activator of FgCdc42 in *F. graminearum*. Rac1 is a Rho GTPase that closely related to Cdc42, which is not appeared in budding yeast but filamentous fungi. Besides, Rac1 and Cdc42 share overlapping functions in some filamentous fungi (Virag et al., 2007; Araujo-Palomares et al., 2011; Kwon et al., 2011; Zhang et al., 2013). Surprisingly, FgCdc42 but not FgRac1 can interact with FgBud3 (**Figure 6B**). Both *FgCDC42* deletion mutant and *FgRAC1* deletion mutant revealed serious growth and conidiation defects (Zhang et al., 2013). However, deletion of *FgCDC42* but not *FgRAC1* led to conidia with a serious abnormal shape which is a similar phenotype of a *FgBUD3* deletion mutant (Zhang et al., 2013). It implied FgBud3 also activated FgCdc42 to regulate morphogenesis of conidia. Rho1 was an upstream regulator of the well known cell wall integrity MAPK pathway in budding yeast (Levin, 2005). FgBud3 did not interact with FgRho1 indicating FgBud3 regulated cell wall integrity not through the potential FgRho1-MAPK pathway in *F. graminearum*. FgRho2 and FgRho4 were important to cell wall integrity (Zhang et al., 2013). The interaction between FgBud3 and FgRho2 or FgRho4 suggested FgBud3 could be a GEF of both FgRho2 and FgRho4 to regulate cell wall integrity.

As we have mentioned above, the *FgBUD3* deletion mutant showed almost the same phenotype as the *FgRHO4* deletion mutant, however, we still found a difference in septum formation between them. In rare cases, conidia with one or even two septa were generated in the *FgBUD3* deletion mutant but not in the *FgRHO4* deletion mutant (**Figures 3A,B, 4A**) (Zhang et al., 2013). One possible reason behind this difference could be attributed to a residual natural capacity in the exchange of GDP to GTP without activation by RhoGEF proteins. The other possible reason could be the existence of another putative RhoGEF protein for FgRho4. In *N. crassa*, two Rho4-specific Rho GEF proteins, Bud3 and Rgf3, are required at different stages of the septation process (Justa-Schuch et al., 2010). We thus speculate that a Rgf3-homolog (gene number, FGSG_08568) could be the other FgRho4 GEF and might also contribute to septum formation in *F. graminearum*. RhoGEF interacts with GDP-bound Rho GTPase to perform the exchange GDP for GTP. In this study, FgBud3 did not only interact with GDP-bound but also with GTP-bound FgRho4. These findings implied different Rho4GEF guided the activated FgRho4 to different targets that may be important for different functions. RhoGEFs usually contain various domains that may be involved in interactions with different effectors to direct Rho GTPase downstream signaling (Mertens et al., 2003). FgRgf3 was predicted to have two additional domains when compared to FgBud3 indicating FgRgf3 may play a number of different roles (**Figure 1**). Future work is needed to further characterize the role of FgRgf3 and determine whether it is also a Rho4 RhoGEF involved in some other functions in *F. graminearum*.

## Author Contributions

CZ, GW, HL, CR, and ZW: conceived and designed the experiments. CZ, ZL, DH, LS, and HY: performed the experiments. CZ and ZL: analyzed the data. CZ: wrote the paper. CZ, HL, CR, and ZW: originated research leading up to this paper and provided guidance and review.

## Conflict of Interest Statement

The authors declare that the research was conducted in the absence of any commercial or financial relationships that could be construed as a potential conflict of interest.

## Footnotes

1 http://fungi.ensembl.org/Fusarium_graminearum/Info/Index

## Supplementary Material

FIGURE S1 Generation of the *FgBUD3* deletion mutants. **(A)** *FgBUD3* gene locus and gene replacement construct. The *FgBUD3* and *hph* genes are marked with empty and black arrows, respectively. P, Pst I. **(B)** Genomic DNA isolated from mycelia of PH-1, and some transformants were subjected to PCR using primer pairs OF/OR and UAF/H853 marked in **(A)**. **(C)** Total RNA samples isolated from mycelia of PH-1, the *FgBUD3* deletion mutant and the complemented strain were subjected to RT-PCR using the *FgBUD3* gene-specific primer QF/QR (**Supplementary Table S1**), *ACTIN* gene was amplified as positive control. **(D)** DNA gel blots of restriction enzymes marked in **(A)** digested genomic DNA were hybridized with probe marked in **(A)**. PH-1, wild-type strain; (Δ*Fgbud3*, gene *FgBUD3* deletion mutants; Fgbud3-com, complemented strain of *FgBUD3* deletion mutant.

TABLE S1 PCR primers used in this study.
