# Supplementary figures and images for: FgBud3, a Rho4-Interacting Guanine Nucleotide Exchange Factor, Is Involved in Polarity Growth, Cell Division and Pathogenicity of Fusarium graminearum
Source: Front Microbiol. 2018 Jun 7;9:1209. doi: 10.3389/fmicb.2018.01209 (PMC5999796; doi:10.3389/fmicb.2018.01209)

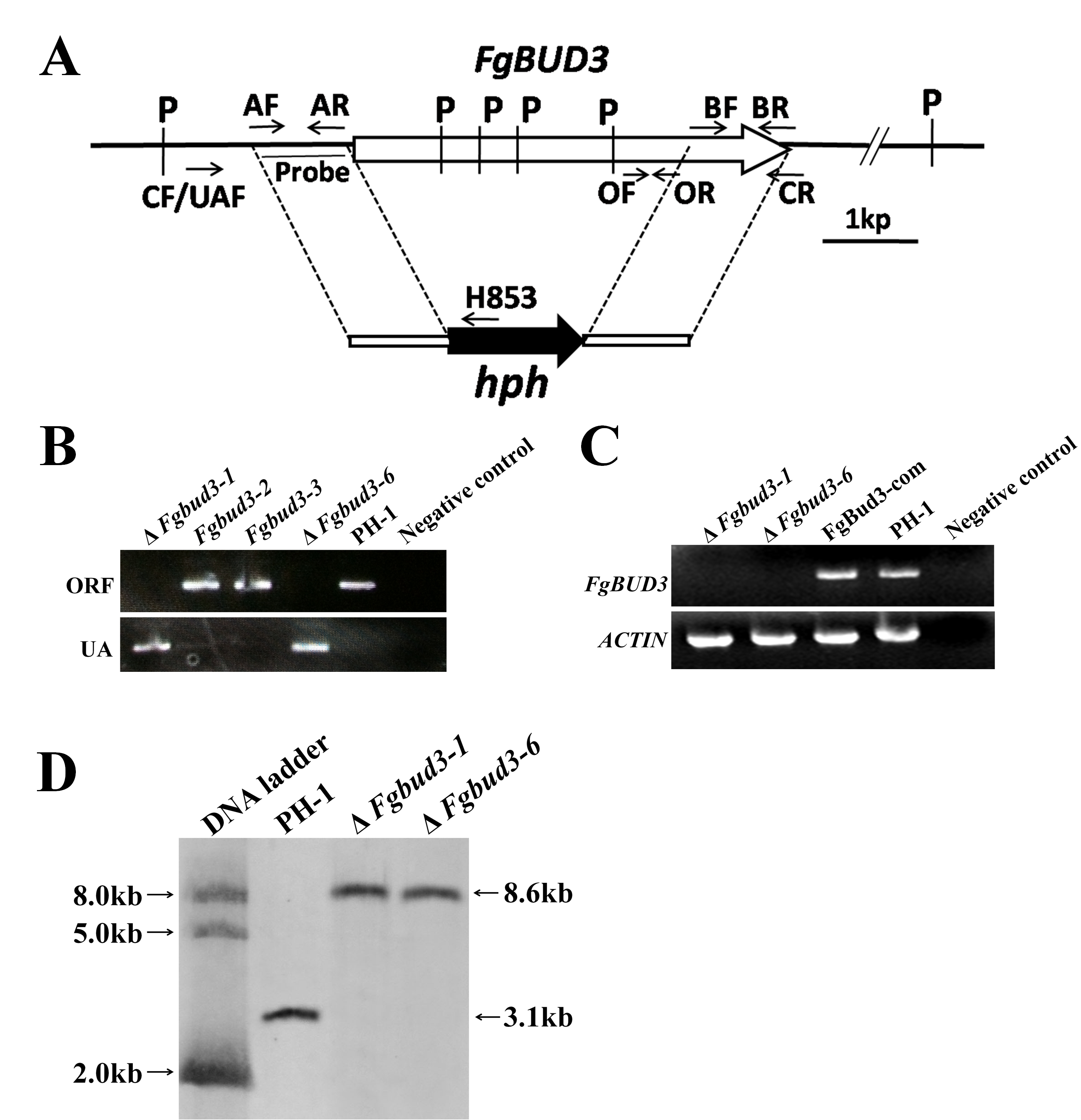

Supplement: FIGURE S1 — Generation of the FgBUD3 deletion mutants. (A) FgBUD3 gene locus and gene replacement construct. The FgBUD3 and hph genes are marked with empty and black arrows, respectively. P, Pst I. (B) Genomic DNA isolated from mycelia of PH-1, and some transformants were subjected to PCR using primer pairs OF/OR and UAF/H853 marked in (A). (C) Total RNA samples isolated from mycelia of PH-1, the FgBUD3 deletion mutant and the complemented strain were subjected to RT-PCR using the FgBUD3 gene-specific primer QF/QR (Supplementary Table S1), ACTIN gene was amplified as positive control. (D) DNA gel blots of restriction enzymes marked in (A) digested genomic DNA were hybridized with probe marked in (A). PH-1, wild-type strain; (ΔFgbud3, gene FgBUD3 deletion mutants; Fgbud3-com, complemented strain of FgBUD3 deletion mutant. [file Image_1.TIF]
